# An analysis of the experiences of bereaved relatives and health care providers following palliative sedation: a study protocol for a qualitative international multicenter case study

**DOI:** 10.1186/s12904-022-01117-w

**Published:** 2022-12-23

**Authors:** M. Van der Elst, S. Payne, M. Arantzamendi, N. Preston, J. Hasselaar, C. Centeno, A. Belar, B. Jaspers, H. Brunsch, S. Surges, C. Adile, J. Menten

**Affiliations:** 1 Department of Oncology, Laboratory of Experimental Radiotherapy, Katholieke Universiteit Leuven, Herestraat 49, 3000 Leuven, Belgium; 2 International Observatory On End of Life Care, Division of Health Research, Faculty of Health and Medicine, Lancaster University, Lancaster, LA1 4AT UK; 3 Institute for Culture and Society-ATLANTES, Universidad de Navarra, Calle Universidad 6, Navarra 31009 Pamplona, Spain; 4 IdISNA- Instituto de Investigación Sanitaria de Navarra. Palliative Medicine, Pamplona, Spain; 5 Department of Anesthesiology, Pain and Palliative Medicine, Radboud University and Radboudumc, Geert Grote Plein 10, HB6500 Nijmegen, Netherlands; 6 Clínica Universidad de Navarra, Palliative Medicine Department, Pamplona, Spain; 7 Department of Palliative Medicine, Universitätsklinikum Bonn, Venusberg Campus 1, 53127 Bonn, Germany; 8 La Maddalena Cancer Center, Via San Lorenzo 312, 90146 Palermo, Italy

**Keywords:** Palliative sedation, Refractory symptoms, Qualitative research, Case study, Relatives, Health caregiver, Study protocol

## Abstract

**Background:**

Patients at the end-of-life may experience refractory symptoms of which pain, delirium, vomiting and dyspnea are the most frequent. Palliative sedation can be considered a last resort option to alleviate one or more refractory symptoms. There are only a limited number of (qualitative) studies exploring the experiences of relatives of sedated patients and their health care professionals (HCPs). The aims of this study protocol are: 1) to elicit the experiences of bereaved relatives and health care professionals of patients treated with palliative sedation and 2) to explore the understanding of the decision-making process to start palliative sedation across care settings in 5 European countries.

**Methods:**

This study protocol is part of the larger HORIZON 2020 Palliative Sedation project. Organisational case study methodology will be used to guide the study design. In total, 50 cases will be conducted in five European countries (10 per country). A case involves a semi-structured interview with a relative and an HCP closely involved in the care of a deceased patient who received some type of palliative sedation at the end-of-life. Relatives and health care professionals of deceased patients participating in a linked observational cohort study of sedated patients cared for in hospital wards, palliative care units and hospices will be recruited. The data will be analyzed using a framework analysis approach. The first full case will be analyzed by all researchers after being translated into English using a pre-prepared code book. Afterwards, bimonthly meetings will be organized to coordinate the data analysis.

**Discussion:**

The study aims to have a better understanding of the experiences of relatives and professional caregivers regarding palliative sedation and this within different settings and countries. Some limitations are: 1) the sensitivity of the topic may deter some relatives from participation, 2) since the data collection and analysis will be performed by at least 5 different researchers in 5 countries, some differences may occur which possibly makes it difficult to compare cases, but using a rigorous methodology will minimize this risk.

**Supplementary Information:**

The online version contains supplementary material available at 10.1186/s12904-022-01117-w.

## Background

Patients at the end-of-life may experience very distressing symptoms of which pain, delirium, vomiting and dyspnea are the most frequent [1]. These symptoms can become refractory because the standard symptom relief is no longer effective or feasible in a sufficient time period, or the risk–benefit ratio is not acceptable to the patient [2]. In such cases, some type of palliative sedation can be an option of last resort [3]. Palliative sedation has been defined differently. Morita et al. formulated in a literature review two core elements of palliative sedation: 1) the presence of severe distress, refractory to standard palliative treatment, and 2) the use of titrated sedative medication with the primary aim of relief of distress by proportional reduction of consciousness [4]. Prior studies indicated that (continuous or intermittent) palliative sedation is a widely used approach at the end-of-life in Europe but with frequency differences between the countries (Denmark 2.5%—Italy 8.5% of deceased patients) [5, 6].

In palliative sedation, various factors are involved such as the prognosis, the indications (physical or non-physical symptoms) and decisions have to be made concerning the mode of application (e.g., the level of sedation, the medication used, the administration route, type of sedation). Previous research shows that the use of palliative sedation differs between countries [7] and varies across clinical settings [5, 8]. For instance, in the Netherlands and Belgium continuous deep sedation is more often applied, while in the UK palliative sedation aims to preserve consciousness as much as possible [7]. One can assume the experiences of relatives and HCPs are also different, but there are few (qualitative) studies to assess the experiences of relatives and health care professionals of sedated patients across care settings and countries [9, 10].

## Horizon2020 Palliative sedation project

The HORIZON2020 project palliative sedation is performed by an international consortium in a five-year project with research teams from eight countries (Radboud UMC (Netherlands), KU Leuven (Belgium), University of Navarra, (Spain), University of Pecs (Hungary), University of Lancaster (United Kingdom), UniversitätsKlinikum Bonn (Germany), Hospice Casa Sperantei (Romania), La Maddalena S.p.a (Italy). This project is coordinated by the researchers of the Radboud UMC (Netherlands).

The main goals of the HORIZON2020 are: 1) To evidence and investigate the practice of proportional palliative sedation using an observational clinical study and a multiple case study 2) To investigate the use of moral case deliberation for palliative sedation 3) To revise the 2009 EAPC recommended framework for palliative sedation 4) To increase public and professional understanding of palliative sedation (see Table 1: the work packages of the Horizon2020 project Palliative Sedation). The European Association of Palliative Care and the European Cancer Patient coalition are also involved in this project to facilitate the dissemination of the results.

**Table 1:** The work packages of the Horizon2020 project Palliative sedation

WP1	Literature review Palliative sedation
WP2	Observational study: praxis and patient comfort
WP3	Case study, interviews with relatives and HCP: experiences and decision-making palliative sedation
WP4	Moral case deliberation palliative sedation
WP5	Cost consequence analysis
WP6	Delphi study: Revision of guideline
WP7	Online educational programme
WP8	dissemination

## The objectives of the present study

1. To elicit the experiences of palliative sedation by bereaved relatives of patients and health care professionals across care settings in five European countries.
2. To explore the decision-making process to start palliative sedation across care settings in five European countries.

## Method

### Design

This is a multiple case study design, exploring a real-life, contemporary phenomenon (palliative sedation) through detailed in-depth data collection involving eliciting multiple sources of information in various care settings and across five EU countries (Belgium, Germany, Italy, Spain, The Netherlands). The qualitative study will consist of semi-structured interviews with a health care professional and bereaved relative 2–3 months after the death of a patient where palliative sedation was implemented. The study will identify 50 cases (10 in each country) recruited from patients who have been treated with proportional palliative sedation (light to deep, intermittent to continuous) in a linked observational cohort study of the HORIZON2020 project (extra information concerning the observational study can be find in supplementary file 1).

### Population and sampling

The present study is sequential to an observational study concerning palliative sedation (WP2), this implies that only relatives and HCP related to patients participating in the observational study will be recruited. The bereaved relative of the patient is defined as a person who was closely involved in the palliative sedation procedure of a patient and is able and willing to speak about her/his experiences. This person will be 18 years or older, speaks the native language of the region but is not necessarily a family member. The health care professional (HCP) will be a physician or nurse involved in the performance of palliative sedation in the particular case. An additional interview (with a relative or a HCP) is recommended if it would add important information to the case, for instance, if a participant ends the interview early. The aim is to build up 50 full cases, meaning an interview with a relative and a health care professional of the patient (in total 100 interviews). A relative/HCP of every patient participating in the observational study will be asked to participate in the present study until we have reached 10 full cases. When a case does not fulfil the criteria of a full case (e.g., relatives or health care professionals decline to participate), a new case will be identified. There is one exemption, if a patient in the observational cohort study has no known relatives (maximum of one such case per country), this exceptional case will be included in the analysis. The recruitment is planned from July 2021 onwards until the beginning of 2023.

#### Relative of the patient

**Fig. 1:**
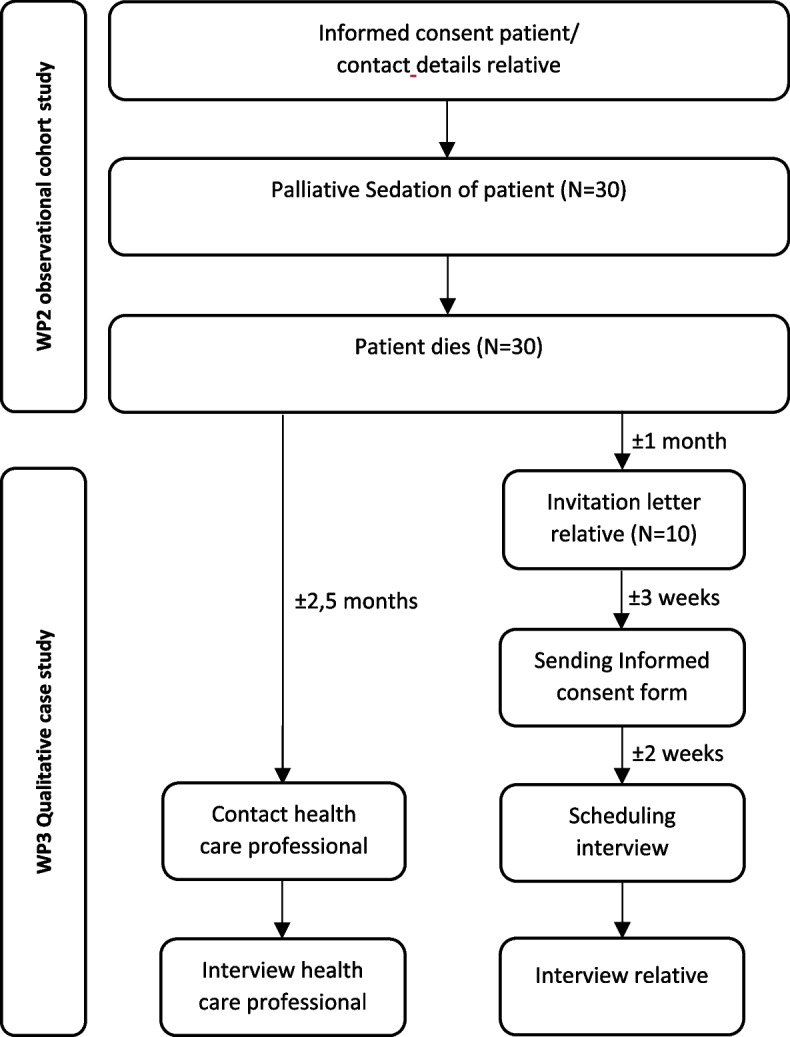
Flow Chart recruitment of participants in each country

If a patient agrees to participate in the observational cohort study (WP2), (s)he will be invited to give the contact details of a relative closely involved in the patient’s care. One month after death, this closely involved relative will receive an invitation to participate in this interview study. The invitation letter will contain the contact details of the researcher, so the patient’s relative can contact the researcher to get extra clarification/information if necessary or to indicate their willingness to participate in the study. Three weeks after the invitation letter, the informed consent form is sent so that the relative will have enough time to reflect and discuss potential participation with a confidant [11]. The relative will be contacted by the researcher a few weeks later to schedule an interview. Approximately three months after the patient’s death, the interview will take place (see Fig. 1: flow chart recruitment of participants). The key principles of the informed consent form will be discussed and signed before the start of the interview.

#### Health care professionals

The health care professionals already aware of the overall project, will be informed about the multiple case study during an information session. The health care professionals closely involved in the palliative sedation of the patient will be invited for an interview to explore their experiences. The key principles of the informed consent will be discussed and the informed consent form will be signed before the interview. They may use the patient’s records, if necessary, to aid recall.

### Data collection

In each country, one or more researchers will perform the interviews and data analysis. To achieve reliable results, interviews and data analysis will be conducted in a consistent way across the different countries. Therefore, several online trainings and consensus meetings will be organized to promote homogeneity in the data collection. During the data collection and analysis, the research teams will work intensively together. Bimonthly online meetings will be held to discuss current problems and to share experiences related to the interviews and data analysis. Via Microsoft Teams, a data collection log will be created where the researchers can discuss issues/experiences that arise during the data collection and analysis to coordinate the working methods and to enhance rigor.

#### Semi-structured interviews

According to Silverman interviewing is an appropriate research method when the aim is to capture a person’s experience, attitudes and motives [12]. The interview format is designed to enable comparison of the data across the various care settings and countries. Therefore, semi-structured interviews are used to explore the participants’ experiences and perspectives. An advantage of semi-structured interviews is the flexible structure, as it gives the participant the possibility to discuss new topics or the interviewer to probe deeper into a specific (new) topic; while the predetermined topic guide ensures the possibility to compare across the cases [13, 14].

#### Interview procedure

Digital recording will be used and the participants will be informed that the recording can be turned off upon request [15]. For the relative of the patient, the interview can take place at the interviewee’s home, unless the interviewee prefers to do this elsewhere (e.g., clinical center). The interviews with the HCP will take place in a private space at the Palliative care unit, hospital ward or hospice. The interview will last approximately one hour for a relative and 30 min for HCPs.

#### Interview topic guide

A case study approach according to Yin benefits from a deductive approach [16]. Therefore, the interview topic guide is based on the EAPC framework for palliative sedation, since this is currently seen as clinical good practice [17]. Robijn and colleagues distinguished four stages of decision-making: initiation, information exchange, deliberation and the decision to start intermittent or continuous palliative sedation until death [18]. Since we are also interested in the decision-making process for palliative sedation, this framework has been also used to develop the topic guide.

A topic guide has been developed for the bereaved relatives (see supplementary Table S1). They will be asked about: 1) how did the idea of palliative sedation arise, what were the (refractory) symptoms 2) how and why palliative sedation was chosen, 3) the procedure used for palliative sedation, 4) the care and interaction of the relatives with the HCPs during the procedure and 5) the care and the interaction of the relatives with the HCPs after the patient’s death and 6) how they experienced the whole process of palliative sedation. 7) the experience of participating an interview about palliative sedation. To facilitate the relative to tell their story about their experiences concerning the sedation of a family member, the interview will always start with the open question: *Can you tell me something about your relative, what were they like?* Afterwards, the interviewer will go through the topic guide, using probes and prompts to elicit more data as required.

A slightly different topic guide has been developed for the HCP (see supplementary Table S2). The HCPs will be asked about a specific patient included in the clinical study regarding: 1) how did the idea of palliative sedation arise, 2) how were the refractory symptoms evaluated, 3) how and why palliative sedation was chosen, 4) the procedure for palliative sedation, 5) the care and interaction with the relatives of the patient during the procedure, 6) the interaction with the relatives and the other HCPs after the patient’s death, and 7) how they experienced the whole process of palliative sedation in the specific case. The interview guide includes probes and prompts and acts as a memory tool, indicating that not every question has to be asked as such. To test the interview guide for its completeness, it was pilot tested with two nurses discussing retrospectively a case. The audio recorded data will be transcribed verbatim by the local investigators and afterwards pseudo-anonymized. The pseudo-anonymized data will be transported into a qualitative software program (e.g., NVIVO). Then the recorded data (audio) will be destroyed. The transcript will be used to analyse the data.

### Covid

Where the COVID pandemic restrictions do not allow us to do in-person interviews, we will use an online interview. If a respondent does not want the interview to take place in person because of COVID, an online or telephone interview will be proposed as an alternative.

### Data analysis

To be able to compare the data across the different applications of palliative sedation, the care settings, and countries; framework analysis is the most appropriate analysis method [19, 20]. This is a matrix-based analytic method that facilitates rigorous and transparent data management so that all the stages involved in the analytic hierarchy can be systematically conducted [19]. It sits within the thematic analysis approach but includes extra steps which facilitate a comparison both within cases in a country and across the cases in the five countries.

The analysis process will be divided into several steps. To promote homogeneity in data analysis, one complete first full case (interviews with a relative and HCP) will be translated into English. This full case will be analyzed by all the researchers involved in this study data analysis. All researchers will code this first full case based on an initial preliminary code book developed after a literature search by the researchers of KU/UZ Leuven and Lancaster team. After the first full case is coded according to the preliminary code book, a meeting will be organized between the researchers to discuss their codings, the preliminary code book, to add or adjust codes, and to optimize the code book by adding additional explanations to the codes and how they can be applied. In the next step, the researchers in each country will undertake the coding of all transcripts (in their original language) based on the adapted code book. During the coding of the transcripts, regular online meetings (every two months) will be held to discuss possible challenges, and to share coding challenges. Via Microsoft Teams, a data analysis log will be created where the researchers can discuss issues/experiences that arise during the data analysis to coordinate the working methods and to enhance rigor. The code book will evolve into a final code book. Once all transcripts are analyzed with this progressively updated code book, the researchers will go through all the interviews to search for issues related to the added topics to the original code book during the online meetings. Afterwards, a summary will be made of each interview and exemplary quotations will be translated into English by the local researcher and sent to the researchers of UZ Leuven. Thereafter the local researchers will do a cross-case analysis within their country. In the last step, the Leuven research team will make a cross-country analysis in consultation with the other research centers.

### Ethical consideration

This study will be in accordance with The Declaration of Helsinki: Ethical Principles for Medical Research Involving Human Subjects [21]. The investigators are aware that this study can be categorized as ‘sensitive research’. Although no agreement exists about the definition of sensitive research; in general it implies any research which has the potential to damage or harms the participant, the researcher, or society [22]. Damage can be physical, but also emotional or psychological. The term ‘sensitive research’ can also be used to describe research involving vulnerable groups such as older people, children, patients with chronic conditions, people with mental health problems, or bereaved ones [22]. To balance the ethical principles of autonomy, beneficence, and non-maleficence, attention will be given to the recruitment procedure, how questions are asked, to the training of interviewers, based on the literature. The participants will be allowed to terminate their participation at any time. Participants are also allowed not to answer questions without any justification or consequences. In case the relative withdraws, the data will be deleted. Relatives and HCP experiencing personal psychological or emotional difficulties after the interview can be supported upon request by a psychologist from the palliative team.

The protocol is approved by the ethical committee of the University Hospital of Leuven (EC Onderzoek UZ/KU Leuven (s63865)) and by the local ethical committees in all country sites. Anonymized and non-traceable data will be made available for re-use by third parties. Although, the dataset will only be partly shared to retain the protection of traceability: only the code book, a synthesis of the transcripts, and data extraction of the analysis will be made accessible after all the scientific reports from this project are published. Paper copies of the ICF (by relatives and health care professionals) will be stored at a locked location in each country to which only the local researchers have access. The signed informed Consent Forms (by relatives and health care professionals) will be digitalized and kept at each participating site adhering to local research data storage policies. The data will be saved in accordance with the local regulations at each site. After this period, data will be destroyed. The informed consent form includes the website of the Horizon2020 Palliative Sedation (EU825700). This website will be kept up-to-date with publications and information about the research project. For those relatives and HCPs interested in the results of the study, a one-page summary with the most relevant results will be prepared. To reassure high ethical standards, also the Guidelines for Conducting Ethical Bereavement Research were followed [15].

## Discussion

This case study will provide information concerning the experiences of bereaved relatives and HCPs and the decision-making process of palliative sedation for refractory symptoms in palliative patients across five European countries. This will shed light on indication and assessment, clinical context, ethical sensitivities, possible distress and uncertainties among caregivers and family members, and communication issues. A case study is an appropriate design since the present study focuses on a contemporary phenomenon and the researcher has no control over the event. A case study design is suitable for exploring practically and ethically complex situations involving a variety of perspectives [16, 23]. Previous research concerning those experiences mostly focused only on one perspective, a health care professional or a relative. Our case study approach enables us to compare the experiences of both and provides a more comprehensive view. The cross-country comparison will shed light on cultural differences in the overall procedure of palliative sedation.

### Strengths

The present design and approach of analysis will allow comparing the experiences of relatives and health care professionals based on the varieties in the application of palliative sedation, setting, and context. This will give an in-depth understanding of the nuances and challenges of end-of-life decisions. Thereby also the data of the observational study can be used to get even a better understanding of the case. Because of the semi-structured nature of interviews, we incorporate flexibility for the interviewer and the interviewee to bring up new topics which are not yet included in the topic guide.

### Operational difficulties

The sensitivity of the topic and interviews occurring during a period of grief may deter some relatives from participating. Consequently, some information can be missed. The interval length (approx. 3 months) between the sedation/death of the patient and the interview can be a limitation. This period can blur the experience, especially for the HCP who sees many patients. Another limitation is that HCP do not work 24/7. The HCP that was active during the decision-making process, is not always the same who is present during the performance of the palliative sedation. HCP are allowed to use the e-health files to mitigate these limitations. We need to be vigilant to claim that the experience and the practice of palliative sedation in our settings are generalizable at the country level. Factors such as the (work-) culture within a setting can have an important impact on the experiences of relatives and HCP, for instance, care setting in or outside the hospital, religious institute versus non-religious, the relation between physicians versus nurses. Since the data collection and -analysis will be performed by at least five different researchers, some heterogeneity could occur during the interviews and the coding process. On the one hand, this could enrich the data with new relevant information, on the other hand, this could make the comparability more difficult between the cases and across the different (care) settings. The heterogeneity will be mitigated by the use of a common topic guide, code book with clarifications, a full case that will be analyzed by all researchers, and by organizing researcher meetings regularly (every 2 months). Conducting research in an international setting poses several special methodological challenges. We hope that the present study protocol will help other researchers to design international qualitative studies.

## Supplementary Information

**Additional file 1: Supplementary material file 1. **Extra information observational study (WP2) HORIZON2020 Palliative sedation.

**Additional file 2: Supplementary Table S1. **Interview guide for bereaved relatives of the patient.

**Additional file 3: Supplementary Table S2. **Interview guide for Health care professionals.

## Data Availability

The datasets generated and/or analysed during the current study are not publicly available due the protection of the traceability but the code trees, the analysis and a synthesis of each transcript will be made accessible for third party re-use on DANS-EASY. Data are however available from the corresponding authors upon reasonable request.

## Abbreviations

EAPC: European Association of Palliative Care
HCP: Health Care Professional
ICF: Informed Consent Form
PCU: Palliative Care Unit
WP3: Work Package 3
UZ and KU Leuven: Universitaire ziekenhuizen and Katholieke Universiteit Leuven
