# Supplementary material for: An analysis of the experiences of bereaved relatives and health care providers following palliative sedation: a study protocol for a qualitative international multicenter case study
Source: BMC Palliat Care. 2022 Dec 23;21:227. doi: 10.1186/s12904-022-01117-w (PMC9783747; doi:10.1186/s12904-022-01117-w)
Supplement: Supplementary file 1 — Additional file 1: Supplementary material file 1. Extra information observational study (WP2) HORIZON2020 Palliative sedation. [file 12904_2022_1117_MOESM1_ESM.docx]

**Supplementary material file 1: Extra information observational study (WP2) HORIZON2020 Palliative sedation**

**Rationale:**

Several studies have been performed about palliative sedation, mostly focusing on continuous deep sedation, with the use of various measurements to monitor its effect. Efficacy of continuous palliative sedation has been monitored by agitation/distress levels, symptom control, levels of sedation/awareness, comfort, safety and family/caregivers satisfaction. Within this observational study, we aim to evaluate the effects on patient comfort of different forms and intensities of palliative sedation: intermittent and continuous, from proportional to deep in a prospective design as part of an international study.

**Objectives:**

To evaluate the effect of palliative sedation on patients’ comfort and other symptoms in different international hospices, palliative care units, and hospital ward settings.

To assess the clinical practice of palliative sedation in different international care settings and the accompanying costs and consequences.

**Study design:** Prospective observational multicenter study in hospices, palliative care units, and hospital ward settings in five European countries (Belgium, Germany, Italy, Spain, and The Netherlands).

**Study sample:**

- The aim is to recruit 30 participants receiving palliative sedation in each country (in total 150)
- Adult patients with advanced cancer and a limited life expectancy.
- Experiencing intractable distress from one or more refractory symptoms (i.e., physical, psychological, and/or spiritual).
- Who is indicated by the clinical team (intention to treat) to receive medication with the aim to reduce consciousness (temporarily or continuously) to relieve suffering?
