# Supplementary material for: An analysis of the experiences of bereaved relatives and health care providers following palliative sedation: a study protocol for a qualitative international multicenter case study
Source: BMC Palliat Care. 2022 Dec 23;21:227. doi: 10.1186/s12904-022-01117-w (PMC9783747; doi:10.1186/s12904-022-01117-w)
Supplement: Supplementary file 2 — Additional file 2: Supplementary Table S1. Interview guide for bereaved relatives of the patient. [file 12904_2022_1117_MOESM2_ESM.docx]

**Supplementary Table S1: Interview guide for bereaved relatives of the patient**

| Table S1: Topic guide and interview guide: relatives of the patient | |
| --- | --- |
| *Topics* | Sample questions: probes and prompts |
| *1. Initiation and information on Palliative Sedation* | -How did the idea of sedation arise?  -What were your initial thoughts about palliative sedation?  -How did you feel when the application of sedative medication was discussed for the first time?  -How did your relative react to this discussion? Was this a relief, made this him/her more anxious?  -Can you tell me more about the things that were discussed during this conversation(s)?  -What information about palliative sedation/end-of-life did you receive?  -What information about palliative sedation/end-of-life did you want/needed? |
| *2. Deliberation and Decision-making process of palliative sedation* | -Can you tell me the reasoning why palliative sedation was chosen?  -Which alternative therapies were discussed (to treat the symptoms?)  -Can you describe how the decision about palliative sedation was made?  -Which persons were involved in the decision-making process and why? |
|  | -Would it be ok if I ask you to tell me how you experienced this period?  -Can you tell me more about the interaction between the family members/relatives of a patient and the health care professionals at this moment? |
| *3. Sedation* | -Would it be ok if I ask you to describe the start and the progress of the palliative sedation of your family member/relative?  -How did you feel during this moment?  -Can you tell me more about the atmosphere during that moment? |
| *4. Monitoring* | -Can you describe the care your family member received by the health care professionals during this period of sedation  -How did you experience this care for your deceased family member/relative?  -Can you tell me something about the symptoms (e.g., pain, dyspnea) during the period your family member/relative was sedated  -How was the interaction with the health care professionals during the sedation of your family member/relative |
| *5. After care* | -Would it be OK if I ask how you experienced the period after your relative passed away?  -After your relative passed away, was there aftercare, and can you tell me more about it? |
|  | -How did you experience this aftercare? |
| *6. Experiences* | -How do you feel after all that process now? |
|  | -Can you tell me If other relatives were involved and how they experienced the palliative sedation of a relative during the whole procedure? |
|  | -What did you miss during the last’s weeks/days of your family member/relative  -What could be improved during this process and how? |
|  | -Looking back, can you tell me what aspects you experienced as difficult during the sedation of your relative?  -Looking back, can you tell me what aspects you experienced as positive during the sedation of your relative? |
| *7. experience interview* | -How did you experience this interview?  -Did you experience this interview as difficult? Can you explain to me why or why not?  -Did you experience some questions as difficult or too emotional?  -What do you think of the timing of the interview? Too early, too late? |
