# Supplementary material for: An analysis of the experiences of bereaved relatives and health care providers following palliative sedation: a study protocol for a qualitative international multicenter case study
Source: BMC Palliat Care. 2022 Dec 23;21:227. doi: 10.1186/s12904-022-01117-w (PMC9783747; doi:10.1186/s12904-022-01117-w)
Supplement: Supplementary file 3 — Additional file 3: Supplementary Table S2. Interview guide for Health care professionals. [file 12904_2022_1117_MOESM3_ESM.docx]

**Supplementary Table S2: Interview guide for Health care professionals**

| Table S2: Topic guide and Interview guide for Health care professionals | |
| --- | --- |
| *Topic list* | Sample questions: probes and prompts |
| *1. Initiation and information on Palliative Sedation* | -Thinking about patient X, how did the idea of sedation arise?  -How would you describe palliative sedation?  -What were your initial thoughts about palliative sedation in the case of patient X?  -When palliative sedation was mentioned the first time with patient X, what was discussed with the patient (relatives of the patient)?  -How did patient X react/respond to the idea of palliative sedation?  -How did the relatives of the patient react/respond to the idea of palliative sedation  -Can you tell me more about the things that were discussed with the family during the initiation of PS  -Which information was requested by the patient/relatives of the patient?  -How was the interaction with the other health care professionals at this stage of the PS |
| *2. Refractory symptoms* | -Thinking about patient X, which refractory symptoms were present?  -Thinking about patient X, how was the assessment of the refractory symptoms done?  -Did the patient suffer existentially? How was this assessed? How did you notice this existential suffering? |
| *3. Deliberation and decision-making of palliative sedation* | -Can you tell me why, in the case of patient X, palliative sedation was chosen?  -Who was involved in the decision-making process?  -How was, in the case of patient X, the decision made?  -What was your role in the decision-making process?  -Were alternative therapies (to treat the symptoms), which? and why was in the end not for this therapy chosen?  -How was the interaction with the family members at this stage of the procedure?  -How was the interaction with the other health care professionals at this stage of the PS?  -How did you experience this decision-making process? |
| *4. Sedation* | -Were you involved in the administering of the patients' sedative medication and can you describe to me what happened?  -Was it difficult to achieve the planned level of sedation? What happened? |
|  | -How did you experience this moment; how did you feel during this administration phase? |
|  | -How did the family/relatives react when the sedative medication was administered? |
| *5. Monitoring* | -To what extent did you monitor patient X during this period of sedation?  -Why or why not was nutrition given?  -Why or why not was hydration given |
|  | -How did you experience the care for the patient during the sedation? |
|  | -Can you tell me something about the course of the refractory symptoms of the patient (e.g., pain, dyspnea) during the sedation  -How, you think, did the family perceive the sedation of their relative  -How was the interaction with the family members during the sedation period |
| *6. Aftercare* | -What happened after the patient passed away?  -Which aftercare (if any) was proposed to the relatives of the patient?  -Which aftercare (if any) was proposed to the healthcare professionals? |
|  | -How was this aftercare experienced? |
| *7. Experiences* | -How did you experience patient X’s palliative sedation?  -Can you tell me how the relatives of the patient experienced the whole procedure of palliative sedation?  -Do you think that the palliative sedation hastened or postponed death, or did not influence the dying of patient X? And why do you think so?  -Did prior experiences influence this case of palliative sedation and how? |
|  | -Looking back, can you tell me what aspects you experienced as difficult during the sedation of patient X? |
|  | -Looking back, can you tell me what aspects you experienced as positive during the sedation of patient X? |
